# Local adaptation in trait-mediated trophic cascades

**DOI:** 10.1098/rspb.2023.2583

**Published:** 2024-01-10

**Authors:** James J. Corbett, Geoffrey C. Trussell

**Affiliations:** Department of Marine and Environmental Sciences and Coastal Sustainability Institute, Northeastern University, Nahant, MA 01908, USA

**Keywords:** trait-mediated, predation risk, foraging, trophic level, plasticity, temperature

## Abstract

Predator-induced changes in prey foraging can influence community dynamics by increasing the abundance of basal resources via a trait-mediated trophic cascade. The strength of these cascades may be altered by eco-evolutionary relationships between predators and prey, but the role of basal resources has received limited attention. We hypothesized that trait-mediated trophic cascade strength may be shaped by selection from trophic levels above and below prey. Field and laboratory experiments used snails (*Nucella lapillus*) from two regions in the Gulf of Maine (GoM) that vary in basal resource availability (e.g. mussels), seawater temperature, and contact history with the invasive green crab, *Carcinus maenas*. In field and laboratory experiments, *Nucella* from both regions foraged on mussels in the presence or absence of green crab risk cues. In the field, *Nucella* from the northern GoM, where mussels are scarce, were less responsive to risk cues and more responsive to seawater temperature than southern *Nucella*. In the lab, however, northern *Nucella* foraged and grew more than southern snails in the presence of risk, but foraging and growth were similar in the absence of risk. We suggest that adaptation to basal resource availability may shape geographical variation in the strength of trait-mediated trophic cascades.

## 1. Introduction

Ecologists have long appreciated the role of predators in shaping community structure and dynamics [1,2]. For example, predators can indirectly benefit basal species through their direct consumption of species in middle trophic levels via a trophic cascade [2–4]. This classical conceptual framework was expanded by studies showing that cascading interactions, and their attendant effects on ecosystems, can also be triggered by predator-induced changes in prey behaviour [5,6] that reduce their vulnerability to predation. Anti-predator behaviours, including reduced foraging time and increased use of refuge habitats, can result in ‘trait-mediated cascades' whose strength can rival that of trophic cascades produced by predator consumption of prey [7–10].

Species residing in middle trophic levels must balance the need to feed with the need to avoid being eaten. This classical ‘foraging–predation risk trade-off’ suggests that ecological processes emanating from the middle of food webs may be just as important as widely appreciated ‘top-down’ or ‘bottom-up’ drivers [11]. Indeed, the effects of the ‘middle-out’ can extend beyond those for community structure and include the transfer of energy through food chains and ecosystem nutrient dynamics [9,11–17]. Importantly, solutions to the foraging–predation risk trade-off can vary among individuals and populations, particularly in cases where there is strong local adaptation in the anti-predator defences of prey. This variation can be shaped by natural selection [18–20] and resulting eco-evolutionary dynamics can unfold across a variety of contexts including the ecological contact history between prey and their predator [18–22].

Efforts to integrate evolutionary thinking into the foraging–predation risk trade-off concept have primarily focused on local adaptation of prey residing in the middle of food chains to their predators above them [18]. Yet, the influence of lower trophic levels (i.e. basal resources) on these dynamics has not received much attention. This neglect may reflect the influence of the life-dinner hypothesis, which posits that selection operates more strongly on anti-predator traits of prey than those related to prey foraging success: the risk of losing one's life is more detrimental to fitness than losing one's dinner [23]. Hence, selection imposed by predators on prey should be stronger than selection imposed by resource effects on prey [23]. However, the role of resources in selection dynamics can have major evolutionary implications, as illustrated by classic work on the linkage between variation in the beak morphology of Galápagos finches and starvation-induced mortality [24,25]. Under benign climatic conditions, finches are able to feed on a variety of food items, but during intense drought large seeds quickly become the most available food source [26]. Because larger, tougher, seeds require bigger beaks to open, finch survivorship during drought is positively correlated with beak size [26]. Hence, in the context of predator–prey interactions, middle species may experience selection from the ‘top’ and the ‘bottom’. Increased attention to how selection from both the top and bottom interact to shape the foraging–predation risk trade-off will become increasingly important because ongoing climate change may alter the structure of natural communities and increase the frequency of extreme weather events that exacerbate the challenges of nutritional stress [27–30].

Predator–prey interactions in the Gulf of Maine (GoM) provide an excellent venue to examine how prey in the middle of food chains solve the risk–foraging trade-off under different selective regimes. The dogwhelk, *Nucella lapillus* (hereafter, *Nucella*) is common throughout the GoM and can strongly influence the structure of rocky shore communities by feeding on mussels (*Mytlius edulis*) and barnacles (*Semibalanus balanoides*) [31–33]. Spatially widespread experiments conducted over the past 20 years have shown that both barnacle and mussel recruitment is dramatically greater in the southern than in the northern GoM [33,34]. Because of these geographical differences in the availability of basal resources, adult *Nucella* in the northern GoM often consume alternative prey, such as limpets and littorinid snails, to compensate for the relative scarcity of barnacles and mussels, but this behaviour is rarely observed in the southern GoM [34]. Even after accounting for alternative species, overall basal resource availability is much greater in the southern than northern GoM [33].

In addition to being important consumers on rocky shores, *Nucella* are also preyed upon by the invasive green crab, *Carcinus maenas* [35,36]. Although the green crab has only recently (last 20 years) become established in the northern GoM, it first invaded the southern GoM in the early 1900s and has been common in this region for at least 100 years [37,38]. Past work has shown that exposure to water-borne risk cues from crabs can induce strong anti-predator responses (both behavioural and morphological) in *Nucella* [9,36,39]. For example, in the presence of green crab risk cues, *Nucella* from the southern GoM exhibit reduced foraging, growth and growth efficiency compared with conspecifics raised in the absence of risk cues [9,36]. Given the latitudinal invasion history (south to north) of green crabs in the GoM and associated variation in selection pressure, the results of studies on southern GoM *Nucella* populations may not apply for populations across their northwest Atlantic range. This discrepancy may be especially evident in the northern GoM where *Nucella* have a shorter contact history with the green crab [38,40] and inhabit a food-poor environment because of the relative scarcity of barnacles and mussels [33,34]. To explore these issues, we conducted a field experiment and a common garden laboratory experiment to examine how *Nucella* populations from the northern and southern GoM vary in their solutions to the foraging–predation risk trade-off.

## 2. Material and methods

### (a) Field experiment

We conducted a field experiment in the northern and southern GoM with four replicate populations within each region (total N = 8 populations; electronic supplementary material, figure S1 and table S1). Juvenile *Nucella* (10.5–13.5 mm in length; mean = 12.08, s.e. ± 0.05) were collected from each population, individually labelled with bee tags, and measured for shell length with digital calipers. Shell and tissue mass were also measured using a non-destructive weighing technique [41]. Four *Nucella* (hereafter response *Nucella*) were then placed in replicate (N = 8 per treatment) plastic ‘response’ chambers (10 × 10 × 7 cm, L × W × H) with 120 juvenile mussels (*Mytilus edulis*) to serve as food. ‘Stimulus’ chambers (10 × 10 × 7 cm, L × W × H) were used to expose *Nucella* to either the presence (Crab) or absence (No Crab) of predation risk (N = 4 replicates for each risk treatment × population combination). Chambers for the Crab treatment received a mature male green crab and four adult *Nucella* to serve as food whereas those for the No Crab treatment (control) received just four *Nucella*. *Nucella* serving as food in the chambers for both treatments were replaced weekly. Pairs of response-stimulus chambers for each population were housed in a larger (14 × 14 × 16 cm, L × W × H) container. In early June 2020, replicate containers for each risk treatment × population combination were placed underneath the fucoid (*Ascophyllum nodosum*) canopy at their native sites. Temperature was monitored every 5 min with Tidbit loggers (Onset Computer Corp.) that were placed within two replicate units at each site. Replicates remained in the field for 28 days, after which the number of mussels consumed in each chamber were counted. All snails were measured for final trait values including shell length, shell mass and tissue mass. Growth was calculated by subtracting initial from final trait values.

*Statistical analyses*. Growth and foraging data were analysed using a two-factor analysis of variance (ANOVA) that considered Region and Risk Treatment as fixed effects and Population as a random effect nested within Region. For growth analyses, replicate containers were a random effect nested within each Risk × Population within Region combination; this was not necessary for the *per capita* analysis of mussel consumption. Replicates where more than two snails had died were excluded (*N* = 4) from the analyses. Regional comparisons of seawater temperatures during the experiment were analysed with a two-factor ANOVA that considered Region as a fixed effect and Site as a random effect nested within Region. We could not perform an analysis of covariance (ANCOVA) with Region as a categorical factor and seawater temperature as the covariate because seawater temperatures in the northern and southern Gulf were so divergent that they did not overlap, thus violating a key assumption of ANCOVA. Hence, to further explore how predation risk may interact with seawater temperature to influence snail foraging and growth for populations within each region, we conducted ANCOVAs that considered Risk Treatment as a fixed effect and mean seawater temperature for each population during the experiment as the covariate. In addition, for each Risk Treatment we used simple linear regressions to characterize the relationships between snail foraging and growth as a function of mean seawater temperature for each population during the experiment.

### (b) Laboratory experiment

For the laboratory experiment, we collected juvenile *Nucella* (11–13 mm in length; mean = 12.02, s.e. ± 0.03) from three populations in the northern and southern GoM (electronic supplementary material, figure S1 and table S1). *Nucella* were tagged and measured as described above and then placed in mesocosms under ambient seawater conditions at the Northeastern University Marine Science Center, Nahant, MA in mid-August 2019. Each mesocosm (27 × 15 × 5 cm, L × W × H) had two chambers separated by a perforated divider. The ‘response chamber’ (16 × 15 × 5 cm, L × W × H) housed four response *Nucella* and a tile that had been seeded with 120 mussels to serve as a food for foraging *Nucella*. This chamber had a plastic mesh (3.75 × 2.90 mm) roof to permit water flow and four PVC spacers (1 cm high) that raised the tile above the floor of each mesocosm. By elevating the tiles, *Nucella* had the option to either forage in the ‘risky’ environment on top of the tile or take refuge below the tile [9]. The other chamber (11 × 15 × 5 cm, L × W × H) served as the ‘stimulus chamber’ and contained either an adult, male green crab and four adult stimulus *Nucella* to serve as food (Crab) or simply four adult *Nucella* (No Crab). Stimulus *Nucella* were sourced from the same population as the response snails in each mesocosm and were replaced every 3 days. Plastic tubing delivered ambient seawater into the stimulus chamber that then flowed through the perforated barrier into the response chamber. This design prevented physical contact between crabs and snails but allowed for delivery of crab risk cues to response snails housed in the downstream response chamber. Each mesocosm was placed in a larger plastic container (35 × 15 × 15 cm, L × W × H) to prevent water exchange among replicates. At the start of the experiment all response *Nucella* were placed on the top side of the tile and thereafter habitat use (risky versus refuge) was recorded every 3 days for each snail. The average proportion of snails in refuge habitat in each response chamber was calculated for each week [42]. Every 6 days, consumed mussels were removed from each mesocosm and placed in labelled plastic bags. The experiment ran for 36 days, after which mussels consumed in each replicate were counted and response *Nucella* were measured for final trait values. Mussel consumption and growth were calculated as described for the field experiment.

*Statistical analyses*. Growth and foraging data were analysed using a two-factor ANOVA that considered Region and Risk Treatment as fixed effects and Population as a random effect nested within Region. For growth analyses, replicate chambers were a random effect nested within each Risk × Population within Region combination; this was not necessary for the *per capita* analysis of mussel consumption. Replicates where more than two snails had died (*N* = 1) were excluded from analyses. The proportion of snails in refuge habitat was analysed using a mixed effect model (ANOVA) that considered Region, Risk Treatment and Week as fixed effects, Population as a random effect nested within Region, and Replicate chamber as a random effect nested within each Week × Risk Treatment × Population within Region combination.

## 3. Results

### (a) Field experiment

**Figure 1.**
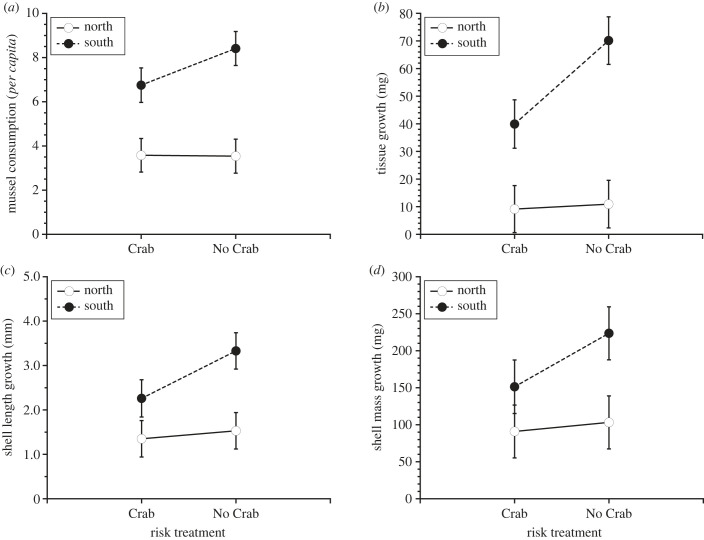
Mean (± s.e.) (*a*) mussel consumption, and growth in terms of (*b*) tissue mass, (*c*) shell length and (*d*) shell mass for snails (*Nucella lapillus*) from the northern and southern Gulf of Maine after 28 days of exposure to the presence (Crab) and absence (No Crab) of green crab (*Carcinus maenas*) risk cues in the field.

*Per capita* mussel consumption varied substantially between regions, with southern *Nucella* consuming significantly more mussels than northern *Nucella* (Region: *F*_1,6_ = 17.7, *p* = 0.0055; figure 1*a*). Surprisingly, we were unable to detect risk effects on mussel consumption (Risk: *F*_1,6_ = 2.38, *p* = 0.174; figure 1*a*; electronic supplementary material, table S3). On average, southern *Nucella* also exhibited more tissue growth than northern *Nucella* (ANOVA, Region: *F*_1,6_ = 16.2, *p* = 0.0068; figure 1*b*). Exposure to green crab risk cues reduced tissue growth (ANOVA, Risk: *F*_1,6_ = 10.9, *p* = 0.0153) but this effect was stronger for southern (–43.1%) versus northern (–16.4%) *Nucella* (ANOVA, Risk × Region: *F*_1,6_ = 8.58, *p* = 0.0248; figure 1*b*; electronic supplementary material, table S3). Exposure to green crab risk cues also reduced shell length growth (ANOVA, Risk: *F*_1,6_ = 10.7, *p* = 0.0165; figure 1*c*) and there was a trend suggesting that the strength of this effect was stronger for southern (–32.1%) than northern (–11.8%) *Nucella* (ANOVA, Risk × Region: *F*_1,6_ = 5.38, *p* = 0.0584; figure 1*c*). Overall, in the field southern *Nucella* grew more in terms of shell length than northern *Nucella* (ANOVA, Region: *F*_1,6_ = 6.07, *p* = 0.0488; figure 1*c*; electronic supplementary material, table S3). On average, shell mass growth did not vary by region (ANOVA, Region: *F*_1,6_ = 3.45, *p* = 0.113; figure 1*d*). Although exposure to risk cues significantly reduced shell mass growth overall (ANOVA, Risk: *F*_1,6_ = 8.55, *p* = 0.0252; figure 1*d*), we were unable to detect a significant interaction with region (ANOVA, Risk × Region: *F*_1,6_ = 4.31, *p* = 0.0812; figure 1*d*; electronic supplementary material, table S3).

**Figure 2.**
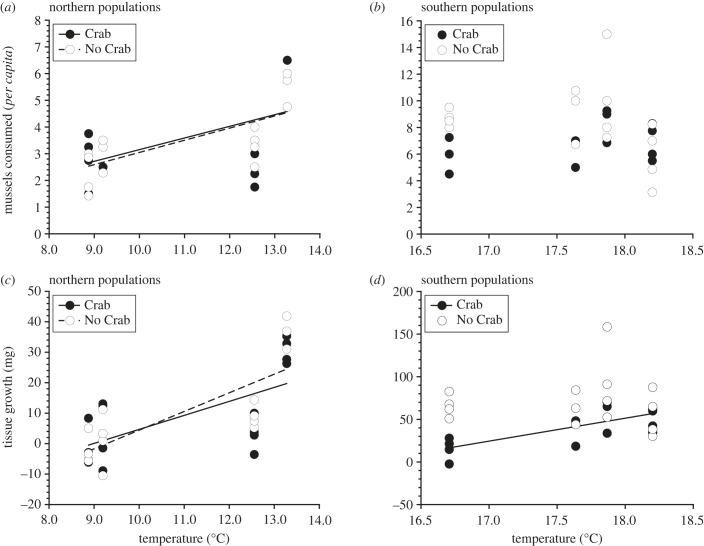
Linear regressions of *per capita* mussel consumption (*a*,*b*) and tissue growth (*c*,*d*) as a function of mean seawater temperature after 28 days of exposure to the presence (Crab) or absence (No Crab) of green crab risk cues in the field. For northern snails, both mussel consumption and tissue growth increased with temperature in the presence and absence of risk cues. For southern snails, we were unable to detect a relationship between mussel consumption and temperature in either risk treatment. Tissue growth in southern snails increased with temperature in the presence of risk. See Results for further details.

The southern GoM averaged 6.62°C warmer than the northern GoM during the experiment (ANOVA, Region: *F*_1,6_ = 31.74, *p* = 0.0013). For northern populations, *per capita* mussel consumption (ANCOVA, Temperature: *F*_1,27_ = 16.9, *p* = 0.0003; figure 2*a*), and growth in terms of tissue mass (ANCOVA, Temperature: *F*_1,27_ = 27.7, *p* < 0.0001; figure 2*c*), shell length (ANCOVA, Temperature: *F*_1,28_ = 44.1, *p* < 0.0001) and shell mass (ANCOVA, Temperature: *F*_1,27_ = 29.0, *p* < 0.0001) all increased linearly with temperature (electronic supplementary material, table S4). For all metrics, we did not detect a significant Risk Treatment effect or Risk Treatment × Temperature interaction (all *p* ≥ 0.40; electronic supplementary material, table S4). Hence, the positive effects of seawater temperature on mussel consumption and tissue growth were similar in the presence (Mussel Consumption—Crab: Y = 0.437X − 1.22, R^2^ = 0.32, *F*_1,14_ = 6.51, *p* = 0.0230; figure 2*a*; Tissue Growth—Crab: Y = 4.60X − 41.3, R^2^ = 0.42, *F*_1,14_ = 10.1, *p* = 0.0068; figure 2*c*) and absence (Mussel consumption—No Crab: Y = 0.456X − 1.51, R^2^ = 0.49, *F*_1,13_ = 12.6, *p* = 0.004; figure 2*a*; Tissue Growth—No Crab: Y = 6.12X − 56.8, R^2^ = 0.61, *F*_1,13_ = 20.0, *p* = 0.001; figure 2*c*) of risk. By contrast, for southern populations, we were unable to detect a relationship between temperature and *per capita* mussel consumption (ANCOVA, Temperature: *F*_1,25_ = 0.0118, *p* = 0.914; figure 2*b*) in either risk treatment (Crab: Y = 1.25X − 15.2, R^2^ = 0.24, *F*_1,12_ = 3.71, *p* = 0.0780; No Crab: Y = 1.09X + 27.6, R^2^ = 0.06, *F*_1,13_ = 0.798, *p* = 0.388; figure 2*b*). There was a trend indicating positive effects of temperature on tissue growth (ANCOVA, Temperature: *F*_1,25_ = 2.98, *p* = 0.097; figure 2*d*) but this was only evident in the presence (Crab: Y = 26.8X − 431.9, R^2^ = 0.51, *F*_1,12_ = 12.5, *p* = 0.0041; figure 2*d*) but not absence of risk (No Crab: Y = 0.767X + 56.4, R^2^ = 0.0002, *F*_1,13_ = 0.0029, *p* = 0.958; figure 2*d*). We were unable to detect a relationship between temperature and growth in terms of shell length (ANCOVA, Temperature: *F*_1,25_ = 1.97, *p* = 0.173) and shell mass (ANCOVA, Temperature: *F*_1,25_ = 1.80, *p* = 0.191; electronic supplementary material, table S4). There was a trend suggesting that exposure to risk reduced *per capita* mussel consumption (ANCOVA, Risk: *F*_1,25_ = 3.86, *p* = 0.0608) and exposure to risk reduced growth in terms of tissue mass (ANCOVA, Risk: *F*_1,25_ = 9.93, *p* = 0.0042), shell length (ANCOVA, Risk: *F*_1,25_ = 8.55, *p* = 0.007), and shell mass (ANCOVA, Risk: *F*_1,25_ = 4.32, *p* = 0.0480; electronic supplementary material, table S4). For all metrics, we did not detect a significant Risk effect or Risk × Temperature interaction (all *p* ≥ 0.11; electronic supplementary material, table S4).

### (b) Laboratory experiment

**Figure 3.**
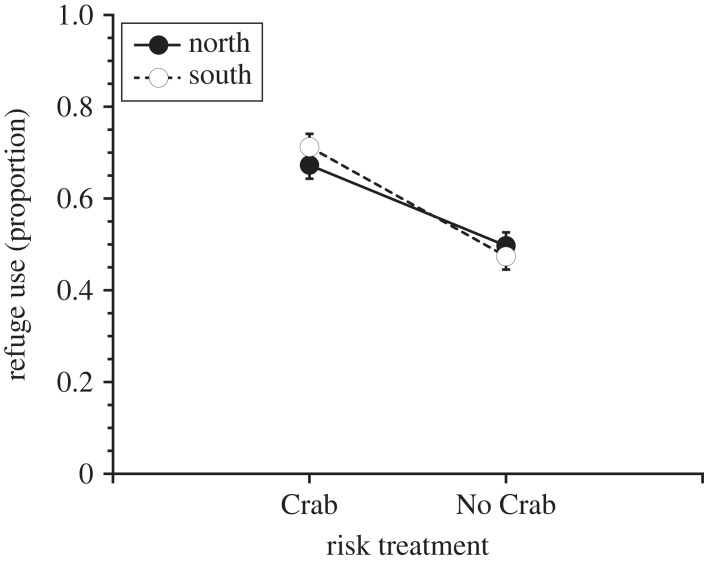
Mean (± s.e.) proportion of time spent in refuge habitat by snails from the northern and southern Gulf of Maine over 28 days of exposure to the presence (Crab) and absence (No Crab) of green crab risk cues in the laboratory.

**Figure 4.**
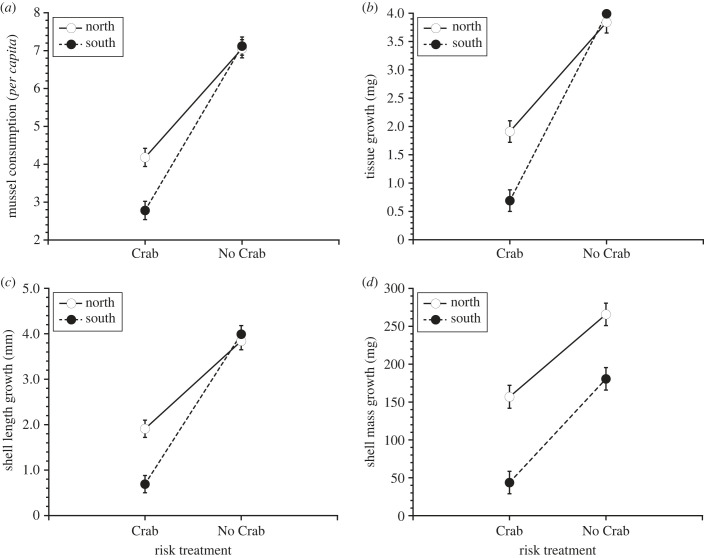
Mean (± s.e.) (*a*) mussel consumption, and growth in terms of (*b*) tissue mass, (*c*) shell length and (*d*) shell mass for snails from the northern and southern Gulf of Maine after 36 days of exposure to the presence (Crab) and absence (No Crab) of green crab risk cues in the laboratory.

*Nucella* exposed to green crab risk cues used refuge habitat more frequently than controls (Risk: *F*_1,4_ = 45.29, *p* = 0.0026; figure 3). Accordingly, exposure to risk cues in the laboratory dramatically reduced *per capita* mussel consumption (ANOVA, Risk: *F*_1,4_ = 365.2, *p* < 0.0001; figure 4*a*), but this effect was stronger for southern (–61%) than northern (–41%) *Nucella* (Risk × Region: *F*_1,4_ = 14.65, *p* = 0.0178; figure 4*a*; electronic supplementary material, table S5). Consistent with the results of our field experiment, *Nucella* exposed to risk cues exhibited large reductions in tissue growth (ANOVA, Risk: *F*_1,4_ = 88.3, *p* = 0.0007; figure 4*b*, electronic supplementary material, table S5), and the strength of this effect varied by region (ANOVA, Risk × Region: *F*_1,4_ = 8.59, *p* = 0.0430; figure 4*b*; electronic supplementary material, table S5) with southern *Nucella* (–84.3%) displaying greater reductions in tissue growth than northern *Nucella* (–62.6%). Unlike the field experiment, we were unable to detect regional differences in tissue growth (ANOVA, Region: *F*_1,4_ = 0.618, *p* = 0.476; figure 4*b*) and shell length growth (ANOVA, Region: *F*_1,4_ = 5.85, *p* = 0.4747; figure 4*c*). However, exposure to risk cues significantly reduced shell length growth (ANOVA, Risk: *F*_1,4_ = 320.5, *p* < 0.0001; figure 4*c*), and the strength of this effect was stronger for southern (–82.7%) than northern (–50.3%) *Nucella* (ANOVA, Risk × Region: *F*_1,4_ = 22.2, *p* = 0.0086; figure 4*c*; electronic supplementary material, table S5). Shell mass growth was substantially different across regions, with northern *Nucella* exhibiting greater shell mass growth than southern *Nucella* (ANOVA, Region: *F*_1,4_ = 28.3, *p* = 0.0059; figure 4*d*). Shell mass growth also decreased with exposure to risk cues (ANOVA, Risk: *F*_1,4_ = 157.2, *p* = 0.0002; figure 4*d*), and *Nucella* from both regions responded similarly (ANOVA, Risk × Region: *F*_1,4_ = 2.05, *p* = 0.23; figure 4*d*; electronic supplementary material, table S5).

## 4. Discussion

It is increasingly clear that community dynamics can be influenced by how species residing in the middle of food chains solve the foraging–predation risk trade-off [11]. Yet, differing eco-evolutionary histories between prey populations and a given predator can yield different solutions to this trade-off, potentially resulting in geographical variation in community dynamics [43]. Recognition that local adaptation to predators can alter how individuals solve the foraging–predation risk trade-off has facilitated conceptual links between evolutionary and community ecology [18], but this perspective has not adequately considered the role of local adaptation to basal resources.

Because of selection imposed by their resource-poor environment, we expected northern snails to display a dampened response to risk cues, but their utter lack of a response in the field experiment was surprising. These results were even more striking when juxtaposed with the risk-induced reductions in growth (tissue mass and shell length, figure 1*b,c*) exhibited by southern snails, which have a much longer contact history with green crabs. These findings contrast with theory predicting that selection favours less plastic, and thus more fixed, phenotypes as the duration of contact history between prey and invasive predators increases [44]. For example, in the late 1990s when the green crab was not well-established in the northern GoM, smooth periwinkles (*Littorina obtusata*) from the northern GoM exhibited greater anti-predator plasticity (increased shell thickening) in response to green crab risk cues than southern conspecifics [40]. Similarly, tadpole populations having no or limited contact history with the invasive red swamp crayfish exhibited greater anti-predator behaviour in response to crayfish cues than tadpoles sourced from populations having consistent historical exposure to this predator [45]. These contrasting results therefore suggest that selection imposed by other factors, such as resource availability, may shape the respective responses of northern and southern *Nucella* to green crab risk cues.

Although risk cues strongly affected the growth of southern *Nucella*, we did not detect an effect of risk on foraging rates in the field (figure 1*a*). The similar levels of mussel consumption exhibited by risk and control snails may reflect the absence of refuge habitat in the response chambers we deployed in the field [46,47]. Under natural conditions in the field, snails typically seek food-poor refugia, such as cracks and crevices, when confronted with predation risk [48–51]. Such refugia were absent in our response chambers and thus may have weakened the foraging–predation risk trade-off. Hence, when given no other option, southern snails foraged at similar rates in the presence and absence of risk cues, but their substantially reduced growth rates in the presence of risk (figure 1*b–d*) suggest that southern snails experienced considerable stress (*sensu* [9]).

We addressed the issue of refuge limitation in the laboratory experiment by incorporating refuge habitat into the stimulus chambers. In the laboratory, both southern and northern *Nucella* used refuge habitat more often when exposed to risk cues (figure 3). As a result, exposure to risk cues significantly reduced mussel consumption in both northern and southern populations (figure 4*a*). Although we did not observe regional differences in behavioural responses (refuge use) to risk cues, the effect of risk cues on *Nucella* tissue and shell length growth and foraging rates differed across the two regions (figure 4*a*–*c*). Consistent with the results of the field experiment, the adverse effects of risk cues on growth were stronger for southern than northern snails (figure 4*b*,*c*). Although we observed similar trends across field and laboratory, there were notable differences with respect to foraging rates and growth between the two experiments (figures 1 and 4). These differences were primarily due to regional effects that manifested in the field experiment (figure 1*a*–*c*). Snails in the southern GoM exhibited significantly greater foraging and growth rates than snails in northern GoM. Additionally, water temperatures experienced by southern snails during the field experiment were much warmer than those experienced by northern snails. Because temperature can strongly influence foraging behaviour, metabolic rate and growth efficiency, it is likely that these differences were shaped by regional thermal regimes [52–54]. Indeed, the positive effects of increased seawater temperature on foraging rates and tissue growth were particularly evident for northern snails regardless of risk treatment (figure 2*a,c*).

In addition to temperature, biotic stressors such as predation risk can have similar effects on organismal physiology by causing increased metabolic rates and reduced foraging and growth efficiency in prey [9,54–57]. Because temperature and predation risk influence performance in similar ways, these two stressors can have interactive effects on a variety of traits [58–60]. For example, plasticity in response to risk cues from predatory dragonfly larvae can strongly interact with temperature to shape life-history traits in *Daphnia magna* populations [58,60]. Our field experiment revealed strong temperature effects on the foraging and tissue growth of northern snails that were similar across both risk treatments (figure 2*a,b*). By contrast, we did not detect temperature effects on these traits for southern snails in the absence of risk, but in the presence of risk there was a trend for increased foraging and clear increases in tissue growth with increasing temperature. These results suggest that for southern snails there may be a threshold temperature above which additional incremental increases in temperature do not matter in the absence of risk, whereas the stress imposed by predation risk favours enhanced leveraging of the postitive effects of increased temperature on foraging and growth.

Although our field experiment revealed regional variation in how prey residing in the middle trophic level respond to predation risk in their native environments, it cannot provide robust insight into the mechanisms driving this variation because northern and southern snails experienced different thermal regimes. Hence, we could not fully parse the effects of risk cues and water temperature in the field. Our laboratory experiment allowed us to explore this issue further, because northern and southern snails were exposed to the presence and absence of risk cues under the same thermal regime (i.e. the warmer water temperatures typical of the southern GoM). Interestingly, the results of the laboratory experiment supported the general trend observed in the field: under common thermal conditions, southern snails still displayed a stronger response to risk cues than northern snails (figure 4*a*–*c*).

Because northern snails experienced warmer water temperatures in the laboratory than they typically encounter in their native environment, their growth and foraging rates may have been influenced by countergradient variation [61]. Countergradient variation can become evident when organisms perform better in other environments relative to their native site [44,62]. Hence, northern *Nucella* may have experienced enhanced growth when maintained under seawater temperatures typical of the southern GoM. If our results were shaped solely by countergradient variation, then we would expect to see its effects in both risk and control treatments [62]. Instead, in the absence of risk we found that mussel consumption was similar for northern and southern *Nucella*, but in the presence of risk northern snails consumed more mussels than southern snails (figure 4*a*). These patterns suggest that selection has favoured less risk-averse behaviour among northern snails perhaps because of the scarcity of preferred food (i.e. barnacles and mussels) in their native environment [33,34]. By contrast, southern snails may be able to engage in more risk-averse foraging behaviour in their native environment because barnacle and mussel recruitment and availability is dramatically higher in the southern versus northern GoM [33].

One might suggest that the lack of a response to risk by northern *Nucella* during the field experiment reflects either a general naivety to green crabs as predators or an inability to detect green crab risk cues (figures 1 and 2*a,c*). This is clearly not the case, because northern *Nucella* responded strongly to green crab risk cues in the laboratory (figure 4). It is also possible that ambient background crab cue may have influenced the results of our field experiment, but previous work [40,51] suggests that these effects are relatively minor because the influence of risk cues was detected even in areas where ambient crab density was high. Indeed, in the current study we again detected a strong response to crab risk cues in the southern GoM (figure 1*b*–*d*). Although green crabs have recently become abundant in the northern GoM, they have been established in the southern GoM for a much longer period of time [37,38]. Given their respective contact histories with the green crab, previous studies imply that northern snails should display a relatively greater response to risk cues even when ambient background cues may be present [40,44,51]. In any case, we suggest that the relatively weak response to risk among northern *Nucella* may reflect selection imposed by the lack of food availability in this region. If persistent low recruitment of barnacles and mussels creates an environment where starvation is a common form of snail mortality, particularly among juveniles who may be too small to consume alternative prey items such as thick-shelled mobile invertebrates (e.g. other molluscs including limpets and littorinid snails), then selection driven by starvation may diminish the influence of risk on solutions to the risk–foraging trade-off in the northern GoM.

We argue that the differences in risk sensitivity among northern and southern populations may reflect selection imposed by geographical differences in food availability, but plasticity (both within and across generations via transgenerational plasticity) in response to water temperature may interact with the effects of predation risk to influence foraging and growth. Such positive temperature effects on foraging and tissue growth operated for northern snails in both the presence and absence of risk, but for southern snails we were only able to detect temperature effects on tissue growth in the presence of risk. Hence, northern and southern snails clearly differ in their responses to the interactive effects of risk and temperature and this may reflect geographical differences in the relative contributions of genetic adaptation and plasticity. Future research that leverages common garden experiments to minimize the effects of environmental history (including maternal effects) will allow a more robust test of this hypothesis.

Geographical variation in the responses of northern and southern snails to predation risk will probably have community-level implications. Trait-mediated trophic cascades, where the non-consumptive effects of predators on prey residing in middle trophic levels can indirectly benefit basal trophic levels, are one of the more notable ways that variable responses to predation risk can affect community structure and dynamics [8,10,19,20,36]. Hence, differences in how *Nucella* from different regions respond to predation risk may influence the relative strength of trait-mediated trophic cascades in the GoM. Seawater temperatures in the laboratory experiment were generally representative of potential future ocean temperature scenarios [63] for the northern GoM, and under these conditions northern snails consumed more mussels than southern snails (figure 4*a*). Hence, the strength of trait-mediated trophic casades in the northern GoM may remain weak relative to the southern GoM with ongoing increases in ocean temperatures under climate change [64]. Because basal resources are scarce in the northern GoM, we suspect that the foraging and growth trends observed in the laboratory under abundant food would not manifest in the field. Such resource scarcity coupled with heightened metabolic demands associated with warmer temperatures may further enhance selection pressure for less risk-averse behaviour [52]. This scenario suggests that the non-consumptive effects of green crabs may further diminish in the northern GoM, but more work is needed to fully explore how community dynamics in this system may change under future climate scenarios.

Our field and laboratory experiments suggest that prey residing in the middle of food chains from distinct geographical regions solved the foraging–predation risk trade-off differently. In the northern GoM, selection shaped by basal resource availability is probably operating because the risk of starvation may have superseded the mortality risk caused by green crabs. In the southern GoM, abundant food allows snails to forgo foraging under periods of heightened predation risk thereby promoting selection for more risk-averse behaviour. More broadly, because food web diversity is dominated by middle trophic levels (60% of total species, [65]), we suggest that the ‘middle-out’ perspective [11] will be highly applicable in numerous systems. Moreover, inclusion of eco-evolutionary links with basal trophic levels will enhance our understanding of the processes shaping community structure and dynamics, especially as the effects of climate change on individual foraging decisions [60], basal resource availability [66] and predator invasions [67] continue to unfold.

## Data Availability

Data from this study are available from the Dryad Digital Repository: http://doi.org/10.5061/dryad.zw3r228dq [68]. Supplementary material is available online [69].
